# Validation of a blubber-based endocrine pregnancy test for humpback whales

**DOI:** 10.1093/conphys/coy031

**Published:** 2018-06-20

**Authors:** Logan Pallin, Jooke Robbins, Nicholas Kellar, Martine Bérubé, Ari Friedlaender

**Affiliations:** 1 Fisheries and Wildlife Department, Marine Mammal Institute, Hatfield Marine Science Station, Oregon State University, Newport, OR 97365, USA; 2 Center for Coastal Studies, 5 Holway Avenue, Provincetown, MA 02657, USA; 3 Marine Mammal and Turtle Division, Southwest Fisheries Science Center, National Marine Fisheries Service, National Oceanic and Atmospheric Administration, 8901 La Jolla Shores Drive, La Jolla, CA 92037, USA; 4 Groningen Institute of Evolutionary Life Sciences, University of Groningen, Nijenborgh 7, 9747 AG Groningen, the Netherlands; 5 Department of Ecology and Evolutionary Biology, Institute for Marine Science, University of California Santa Cruz, 115 McAllister Way, Santa Cruz, CA 95060, USA

**Keywords:** Biopsy, blubber, humpback whale, life history, pregnancy, progesterone

## Abstract

Baleen whales have few identifiable external indicators of pregnancy state, making it challenging to study essential aspects of their biology and population dynamics. Pregnancy status in other marine mammals has been determined by measuring progesterone concentrations from a variety of sample matrices, but logistical constraints have limited such studies in free-swimming baleen whales. We use an extensive blubber sample archive and associated calving history data to retrospectively identify samples that correspond to pregnant females and develop a progesterone-based pregnancy test for humpback whales. The lowest pregnant blubber progesterone concentration was 54.97 ng g^−1^, and the mean for the known-pregnant group was 198.74 ± 180.65 ng g^−1^. Conversely, females known to be below the minimum age of sexual maturity (juvenile females) had an overall low mean progesterone concentration (0.59 ± 0.25 ng g^−1^), well below the known-pregnant range. Of the mature females that did not return with a calf (*n* = 11), three fell within the known-pregnant range (320.79 ± 209.34 ng g^−1^), while the levels for the remaining eight were two orders of magnitude below the lowest known-pregnant level (1.63 ± 1.15 ng g^−1^). The proportion of females that did not return with a calf but had values similar to known-pregnant females are consistent with rates of calf mortality, but other potential explanations were considered. Our findings support a validated blubber endocrine assignment of pregnancy corroborated with field life history information, a first for any baleen whale species. The progesterone values we measured were similar to those found in different pregnancy states of other cetaceans and support using blubber biopsy samples for assigning pregnancy in humpback whales. This method can be applied to existing archives or new samples to better study life history and population demography broadly across species and populations.

## Teaser

There are few identifiable external indicators of pregnancy state in live baleen whales. However, progesterone can be quantified from biopsy samples that are collected from free-ranging whales. We use a blubber sample archive and associated calving data from a well-studied population to develop a blubber-based pregnancy test for humpback whales.

## Introduction

Baleen whales are cryptic animals with few identifiable of pregnancy status. These limitations make it challenging to study essential aspects of their biology and population dynamics (Hunt *et al.*, 2013b). Humpback whales are seasonal breeders that migrate between low latitude mating and calving grounds and mid- to high-latitude feeding grounds. Like most baleen whale species, detailed scientific knowledge on humpback whale (*Megaptera novaeangliae*) reproduction initially came from scientific observers in commercial whaling programs who examined the reproductive tracts of freshly killed humpback whales, noting evidence of pregnancy (presence of foetus or corpora) (Chittleborough, 1954, 1955, 1965). Most cetaceans, including humpback whales, appear to be seasonally polyestrous (Chittleborough, 1954; Robeck *et al.*, 2018). For humpbacks, oestrus is believed to begin while on the breeding grounds and terminates when the migration to feeding areas begins (Chittleborough, 1955; Matthews, 1937). Pregnant females undergo a return migration to the breeding ground late in the gestational term the following winter to give birth after an 11–12-month pregnancy (Chittleborough, 1958). Female humpbacks reach sexual maturity between 4 and 5 years of age (Chittleborough, 1965) and have average inter-calf intervals of 2.3 years (Clapham and Mayo, 1990). Since the cessation of commercial whaling in the 1980s, data on humpback whale reproduction has come primarily from long-term studies of individual females and their calving histories (Barlow and Clapham, 1997; Clapham and Mayo, 1987; Gabriele *et al.*, 2017; Glockner-Ferrari and Ferrari, 1990; Herman *et al.*, 2011; Robbins, 2007).

Long-term observational studies provide valuable, although limited, data on reproduction because observational data only detects those pregnancies that yield surviving offspring. They also depend on longitudinal datasets that are not feasible for many populations. Knowledge of both pregnancy rates and calving rates provides greater understanding of population health and potential for growth. Direct data can also be obtained from stranded animals, but these are relatively rare events, typically involving degraded specimens. In addition, the individuals involved may not be representative of the entire population (Iwasaki, 1997). Our knowledge of pregnancy in baleen whales is still primarily informed by whales killed more than a decade ago, including those drawn from different populations and influenced by different population dynamics than experienced by whales today. Reliable techniques are therefore needed to accurately assign pregnancy status to live baleen whales in order to improve our understanding of their biology, population dynamics and recovery status.

Progesterone, often referred to as the hormone of pregnancy, is a lipophilic circulatory sex steroid hormone produced by corpus luteum (CL) and is the primary progestogen source necessary for the establishment and maintenance of pregnancy (Pineda, 2003). In marine mammals, pregnancy status in live animals has been determined by measuring the concentration of steroid hormones from a variety of biological matrices across multiple species (Atkinson *et al.*, 1999; Pietraszek and Atkinson, 1994; Walker *et al.*, 1988; Wells *et al.*, 2014; West *et al.*, 2002). To date, these methods have had limited application to baleen whales which because of their size, lack of captive specimens and relative scarcity make such methods impossible. Sex steroids have been studied in faecal and breath mucosa samples from free-swimming North Atlantic right whales (Hunt *et al.*, 2013a, 2014a; Rolland *et al.*, 2005), but such samples are not always available in adequate quantities or on demand from the individuals of interest.

Progesterone can be reliably quantified from cetacean blubber (Kellar *et al.*, 2006; Mansour *et al.*, 2002; Trego *et al.*, 2013), and small quantities of blubber can be obtained from live whales using remote biopsy techniques (Palsbøll *et al.*, 1991). Biopsy sampling is already widely used to obtain skin for molecular genetic studies and blubber tissue is often obtained simultaneously. A few studies have attempted to assess the pregnancy state of live cetaceans from biopsy-based blubber (Clark *et al.*, 2016; Kellar *et al.*, 2006; Pérez *et al.*, 2011; Trego *et al.*, 2013), but none have ground-truthed these findings with samples from individuals of known pregnancy status in large baleen whales. Rather, they have assigned pregnancy to individuals by developing a threshold based on the distinctly higher levels of progesterone associated with being pregnant (Clark *et al.*, 2016; Kellar *et al.*, 2006; Pérez *et al.*, 2011; Trego *et al.*, 2013). Assigning such a threshold in biological populations is problematic in light of the likelihood that progesterone values vary to some degree among individuals (Clark *et al.*, 2016; Kellar *et al.*, 2006).

Extensive life history datasets combined with biopsy progesterone-based pregnancy assignments offer a unique opportunity to assess both the pregnancy state and calving outcome from the same individual females. The Gulf of Maine, off the east coast of North America, is one of the primary humpback whale feeding areas in the North Atlantic. Female humpback whales that feed in the Gulf of Maine mate in the West Indies in winter, with peak breeding around February. Individual humpback whales have been studied on this feeding ground since the 1970s, resulting in a well-established catalogue of known individual females and their calving histories, as well as an extensive archive of tissue samples. The goal of this study was to use an extensive archive of blubber samples and associated calving data from this well-studied population to develop and validate a blubber-based pregnancy test for free-swimming humpback whales.

## Materials and methods

### Sample collection

We obtained blubber samples between April and November (1999–2015) using standardized remote biopsy techniques (Palsbøll *et al.*, 1991). Samples were obtained from the upper flank below the dorsal fin and sampled individuals were individually identified from their natural markings, especially the ventral pigmentation of the flukes and the shape and size of the dorsal fin (Katona and Whitehead, 1981). We obtained demographic and life history data for sampled females from the Gulf of Maine Humpback Whale Catalogue curated by the Center for Coastal Studies (Massachusetts, USA). Sex was determined from a molecular genetic analysis of skin samples (Bérubé and Palsbøll, 1996; Palsbøll *et al.*, 1992), the external morphology of the genital slit (Glockner and Venus, 1983) or both. The pregnancy state of females was categorized based on available life history data to evaluate the results of progesterone assays.

### Field pregnancy assignment

Control samples were established from females of known pregnancy status. “Pregnant” females were known to have been pregnant at the time of sampling because they were re-sighted with a dependent calf in the year after they were sampled. Dependent calves were classified in the field by their close proximity and consistent association with a single animal at least twice their size. They exhibited stereotypical positioning and behaviours not observed in older animals and photo-identification confirmed that they were new to the catalogued population. They were assumed to range from 3 to 9 months old when first observed and typically remained dependent until at least October of their first year (Baraff and Weinrich, 1993; Clapham and Mayo, 1987).

“Juveniles” were specifically included in the study because we were interested in predicting the probability of pregnancy in female humpback whales from populations where no demographic (e.g. age structure, maturity, calving histories) information is known. They also served as a negative control in this study because they were known to be younger than the minimum age at sexual maturity. They were first catalogued as dependent calves and known to be 1–2 years of age, whereas the minimum age of sexual maturity in this species is between ages four and five (Chittleborough, 1965; Clapham, 1992; Robbins, 2007).

In addition to controls, samples from other females were analyzed to further describe the range of progesterone concentrations in female humpback whales. These samples were taken from females who were re-sighted without a calf the following year, but known to be mature because they had a prior calving history (“Resting”) and/or a dependent calf when sampled (“Lactating”). While they were not seen with a calf the following year, they could have simply experienced a failed pregnancy after sampling, had a calf that suffered neonatal mortality before arrival at the study area, or experienced a reproductive anomaly (e.g. pseudopregnancy). It was not possible to differentiate among these possibilities from available life history data. However, results from these samples nevertheless served to further establish the range of progesterone values in mature animals from the same population.

### Hormone extraction

Blubber samples were stored frozen at −20°C until analysis. Methods to extract hormones from skin and blubber biopsy samples followed those described by Kellar *et al.* (2006) and Trego *et al.* (2013). In short, the blubber portion of the biopsy sample was sub-sampled (~0.15g) spanning the entire depth of the sampled blubber layer. It is relevant to mention that these samples do not represent the full depth of the blubber layer, only the outermost 10–30mm. The blubber was then homogenized in ethanol using an automated, multitube homogenizer (Bead Ruptor 12, Omni International). The resulting homogenate was then run through a series of chemical washes and separations, lastly providing a final hormone extract that was frozen until assayed (Trego *et al.*, 2013).

Progesterone concentrations (ng progesterone g^−1^ blubber; ng g^−1^) were quantified using a progesterone enzyme immunoassay (EIA; Enzo Life sciences, kit ADI-900-011). Prior to analysis, samples were re-suspended in 1 ml of phosphate buffered saline (PBS; pH 7.5), containing 1% bovine serum albumin and vortexed thoroughly (Kellar *et al.*, 2006). Two additional standard dilutions were added to allow for a lower detection limit of the standard curve to 3.81pg/ml. Samples were run blind and in duplicate. If a sample failed to fall within the detection limit of the assay curve, the sample was re-run at varying dilutions. The reported inter-assay coefficient of variation (COV) and intra-assay COV of the progesterone EIA kit ranged from 2.7 to 8.3% and 4.9 to 7.6%, respectively. Additionally, strong assay parallelism has been show in blubber samples from this species elsewhere (Clark *et al.*, 2016; Pallin *et al.*, 2018).

### Model development

We developed a simple binomial logistic regression model in MATLAB to model the probability of a female humpback whale being pregnant as a function of quantified blubber progesterone (BP) concentrations (Kellar *et al.*, 2017). Progesterone concentrations were used as the predictor variable. All BP concentrations were log transformed prior to model development. The model output generated probabilistic estimates of pregnancy for each female input into the model. Lastly, we estimated the 95% confidence envelope associated with each probability by bootstrap resampling, with replacement, 10 000 times across the range of potential hormone values. The bootstrapping matrix was then sorted and the lower 2.5% and upper 97.5% envelopes were called from the 250th and 9750th iteration.

### Pregnancy determination

All samples were classified as either pregnant or not-pregnant in relation to the range of progesterone results obtained from known-pregnant females. We hypothesized that the mean progesterone values from females that returned with a calf would be significantly greater than the mean concentrations of both females that did not return with a calf and juvenile females.

### Model application to unknown populations

To demonstrate the use of this model in determining the probability of pregnancy of biopsied female humpback whales of unknown pregnancy status, we selected 11 females (from an established sample archive as part of a long-term ecological study by the authors) sampled along the Western Antarctic Peninsula (WAP) in 2013–2016 (Table 2;Pallin *et al.*, 2018). These samples were specifically selected from available values to illustrate how this methodology can be used to estimate the probability of pregnancy in unknown animals across the possible range of BP concentrations. As such, having selected specific values from unknown individuals allows for a more thorough discussion about how to interpret a set of empirical values that are likely to occur. Consequently, the resulting proportion of pregnant, non-pregnant and unassigned animals from this specific exercise should not be assumed to be representative of the WAP population sample.

Based on the progesterone concentrations from these samples, the model determined the probability of pregnancy (point estimate) and 95% confidence envelope. Using both the point estimate and associated error, we were then able to definitively assign pregnancy (e.g. >99.9% is pregnant, <0.1% non-pregnant; Kellar *et al.*, 2017). Moreover, we were also able to provide an estimate of the proportion of pregnant females that included all samples, including those with an assignment probability between 0.1% and 99.9%, and incorporated an appropriate level of uncertainty around the estimate. This was accomplished by taking the sum of the probabilities for all samples at each individual bootstrap replicate and dividing by the sample size to obtain the proportion pregnant at each bootstrap. These proportions were then sorted, and the median estimate and 95% confidence envelope were calculated.

## Results

### Field-observed reproductive state versus blubber progesterone

Females that returned with a calf (*n* = 12) exhibited high average progesterone concentrations (mean = 198.74 ± 180.65 ng g^−1^; Table 1), including two samples collected simultaneously from the same female that both yielded elevated progesterone concentrations (119.5 and 171.78 ng g^−1^). Pregnancies leading to a viable calf were successfully detected based on progesterone concentrations alone, regardless of the timing of the sampling on the feeding ground (April–December, Table 1).

**Table 1:** Field-observed and endocrine assignments of pregnancy from female humpback whales biopsied in the Gulf of Maine^1^

Sample ID	Age	Age type	Sample date (dd.mm.yy)	Progesterone (ng g^−1^)	log_10_(P4)	*P*_Pregnant_ (%)	Lower CI (%)	Upper CI (%)	Endocrine pregnancy assignment	Field pregnancy assignment	Reproductive outcome	Storage (years)
CCS2009-056	1	Exact	3-08-09	0.20	−0.69	0.00	0.00	0.00	No	Juvenile	1	7.31
CCS2015-082	1	Exact	9-11-15	0.50	−0.3	0.00	0.00	0.00	No	Juvenile	1	1.04
CCS2015-011	33	Minimum	26-04-15	0.54	−0.26	0.00	0.00	0.00	No	Lactating	2	1.58
CCS2009-099	1	Exact	19-11-09	0.55	−0.26	0.00	0.00	0.00	No	Juvenile	1	7.01
CCS2009-073	2	Exact	14-08-09	0.66	−0.18	0.00	0.00	0.00	No	Juvenile	1	7.28
CCS2015-067	17	Exact	31-08-15	0.69	−0.16	0.00	0.00	0.00	No	Resting	2	1.23
CCS2010-098	2	Exact	19-10-10	0.71	−0.15	0.00	0.00	0.00	No	Juvenile	1	6.10
CCS2009-095	1	Exact	14-09-09	0.94	−0.03	0.00	0.00	0.00	No	Juvenile	1	7.19
CCS2015-076	28	Minimum	18-09-15	0.94	−0.02	0.00	0.00	0.00	No	Resting	2	1.18
CCS2009-076	21	Minimum	16-08-09	1.35	0.13	0.00	0.00	0.00	No	Lactating	2	7.27
CCS2005-009	27	Minimum	20-06-05	1.46	0.17	0.00	0.00	0.00	No	Lactating	2	11.43
CCS2011-063	36	Minimum	2-08-11	1.63	0.21	0.00	0.00	0.00	No	Resting	2	5.31
CCS2008-110	29	Minimum	13-11-08	2.35	0.37	0.00	0.00	0.00	No	Resting	2	8.03
CCS2004-029	19	Exact	26-07-04	4.09	0.61	0.00	0.00	0.00	No	Lactating	2	12.33
CCS2011-021	11	Exact	20-06-11	54.97	1.74	100	100	100	Yes	Pregnant	3	5.43
CCS2006-026	15	Exact	30-07-06	65.11	1.81	100	100	100	Yes	Pregnant	3	10.32
CCS2013-008	26	Minimum	7-08-13	74.5	1.87	100	100	100	Yes	Pregnant	3	3.30
CCS2008-013	21	Exact	27-04-08	95.33	1.98	100	100	100	Yes	Pregnant	3	8.58
CCS2008-112	25	Exact	27-06-08	107.59	2.03	100	100	100	Yes	Pregnant	3	8.41
CCS1999-092*	24	Minimum	20-08-99	108.11	2.03	NA	NA	NA	Yes	Lactating	2	17.27
CCS2015-079	27	Exact	4-11-15	119.5	2.08	100	100	100	Yes	Pregnant	3	1.05
CCS2007-111	22	Minimum	9-11-07	146.18	2.16	100	100	100	Yes	Pregnant	3	9.04
CCS2015-080	27	Exact	4-11-15	171.78	2.23	100	100	100	Yes	Pregnant	3	1.05
CCS2007-010	16	Exact	24-05-07	174.51	2.24	100	100	100	Yes	Pregnant	3	9.51
CCS2008-033	30	Minimum	27-06-08	245.62	2.39	100	100	100	Yes	Pregnant	3	8.41
CCS2015-069*	15	Exact	6-09-15	327.66	2.52	NA	NA	NA	Yes	Resting	2	1.21
CCS2011-026	11	Exact	10-07-11	513.48	2.71	100	100	100	Yes	Pregnant	3	5.38
CCS2015-075*	24	Minimum	28-09-15	526.62	2.72	NA	NA	NA	Yes	Resting	2	1.15
CCS2007-019	17	Exact	24-05-07	616.36	2.79	100	100	100	Yes	Pregnant	3	9.51

^1^Progesterone concentrations are reported as the ng progesterone g^−1^ blubber (ng g^−1^). Endocrine pregnancy status refers to the pregnancy assignment based on the progesterone concentrations and logistic model output. Field pregnancy assignment refers to the field-observed reproduction status of the individual female when she was re-sighted the following field season. For example, a “Lactating” status refers to a female that was biopsied accompanied by a calf and observed without a calf the following field season. “Resting” refers to a female that was not accompanied by a calf when biopsied nor was it accompanied by a calf the following field season. “Pregnant” refers to a female that was biopsied and was either accompanied by a calf or not and observed with a calf the following field season. Reproductive outcome designates the combined assessment of both endocrine and field pregnancy assignments. In the reproductive outcome column, 1 = females below the minimum age at sexual maturity (juvenile), 2 = females that did not return with a calf and 3 = females that returned with a calf. *Females with progesterone concentrations consistent with pregnancy that did not return with a calf.

Mature females that did not return with a calf the following field season (*n* = 11) had progesterone concentrations that fell both within and outside of the known-pregnant range. Three of 11 (27%) females had progesterone concentrations similar to known-pregnant females (mean = 320.80 ± 209.34 ng g^−1^; Table 1) even though they did not have a calf when re-sighted the following field season. This was not unexpected; in the wild pregnant mammals often lose offspring either during pregnancy or during the first months of life. These females had been sampled in the second half of the feeding season when they were potentially six or more months pregnant, but still possibly vulnerable to neonatal mortality and possibly late term pregnancy failure. However, given that we could not confirm the pregnancy status of these females, they were excluded from statistical analyses and the model development. The remaining 8 of 11 (73%), females were classified as not-pregnant for model development because their low progesterone concentrations (mean = 1.63 ± 1.15 ng g^−1^; Table 1) were outside of the known-pregnant range. They had, on average, two orders of magnitude lower progesterone concentrations than known-pregnant females (unpaired two-sample *t*-test, two-sided *P*-value = 0.007, *df* = 18, Table 1).

**Figure 1:**
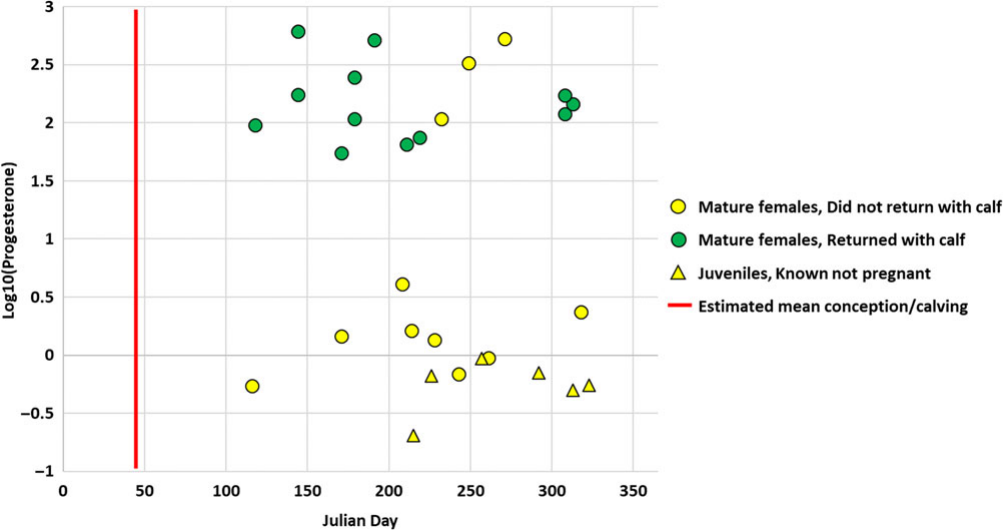
Variation in progesterone concentrations (ng g^−1^) and reproductive outcome of female humpback samples from the Gulf of Maine. Shapes correspond to age class and colours correspond to reproductive outcome. The red line represents the estimated mean conception/calving date which occurs on Julian Day 46 (February 15). Note: high progesterone concentrations are generally indicative of pregnancy.

**Figure 2:**
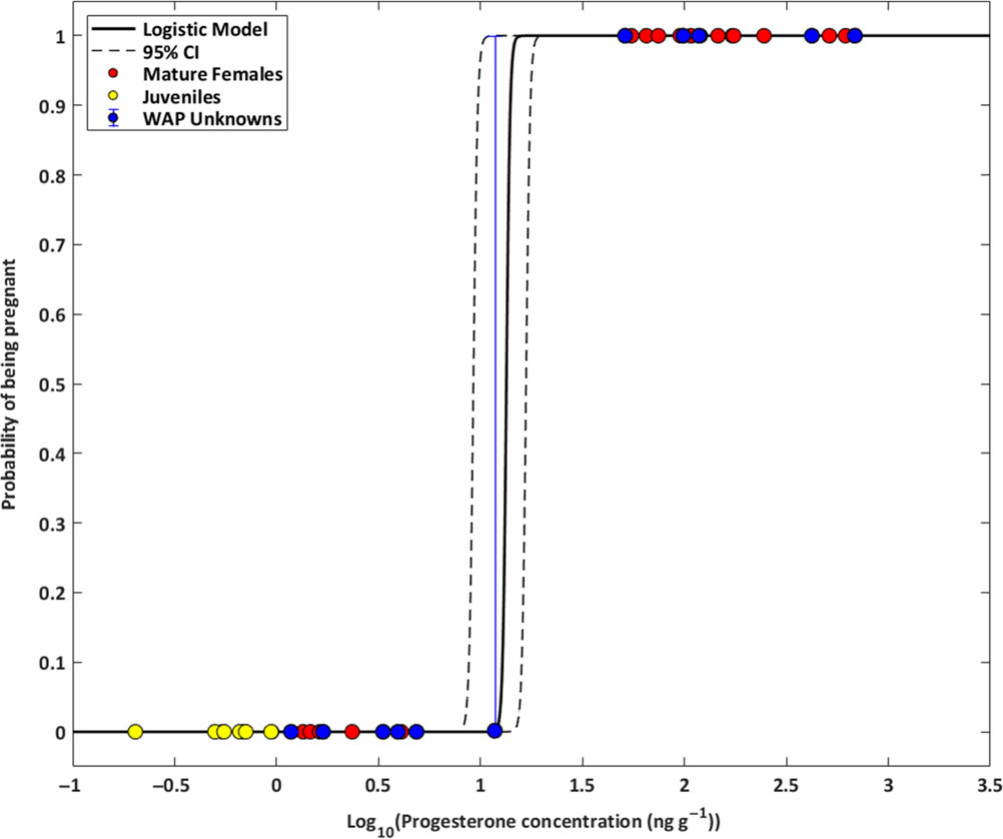
Logistic regression model for the probability of pregnancy in humpback whales relative to blubber progesterone concentration. Red circles represent mature females from the Gulf of Maine, excluding three for which pregnancy testing and calving data yielded inconsistent results. Yellow circles represent known juveniles from the Gulf of Maine. Blue circles represent the selected 11 females of unknown pregnancy status sampled along the Western Antarctic Peninsula with associated error around their probability of pregnancy. Dashed lines represent the 95% confidence envelopes developed from 10 000 bootstrap iterations. *x*-axis values are log_10_ transformed.

Juvenile females (*n* = 6) that could not have been pregnant had a low mean progesterone concentration (0.59 ± 0.25 ng g^−1^), with no false detections and no overlap in progesterone concentration values with females known to have been pregnant. They had significantly lower progesterone concentrations when compared to both known-pregnant females (unpaired two-sample *t*-test, two-sided *P*-value = 0.018, *df*= 16, Table 1) and adults with progesterone concentrations outside of the pregnant range (unpaired two-sample *t*-test, two-sided *P*-value = 0.050, *df* = 12, Table 1). Progesterone concentrations consistent with pregnancy were detected after extended freezer storage, and pregnancy was inferred from one sample that had been frozen for 17.3 years (Table 1). None of the samples analyzed exhibited a progesterone concentration between 5 and 50 ng g^−1^, providing great specificity for assigning pregnancy using the logistic model (Figs 1–2, Table 1). The best fit model (Fig. 2) describing the relationship between pregnancy state and BP concentrations with these humpbacks was: $P_{\mathrm{pregnancy}}=\frac{1}{1+\exp^{-\left(\beta_{0}+\beta_{1}\times \mathrm{BP}\right)}}$ where the mean coefficients and their 95% confidence intervals were *β*_0_ = 113.59 ± 2.25 × 10^7^ and *β*_1_ = −128.01 ± 3.39 × 10^7^.

### Model results for unknown females

When applying the above model to the 11 humpback females biopsied along the WAP, five were estimated to have a higher than 99.9% median probability of being pregnant and five were estimated to have a probability of being pregnant <0.1% (Table 2). Additionally, one individual, whose progesterone concentration fell within the 95% confidence envelope (11.81 ng g^−1^), received a probability of being pregnant of 0.15%, with a lower CI of 0.00% and upper CI of 100%. This individual received an undetermined pregnancy designation. The estimated proportion pregnant, derived from a series of 10 000 bootstrap samples across all 11 samples of unknown pregnancy status, was 45.47% (CI = 45.45–54.54%).

**Table 2:** Endocrine results and pregnancy assignments for eleven chosen female humpbacks of unknown pregnancy status sampled along the Western Antarctic Peninsula

Sample ID	Julian day	Year	Progesterone (ng g^−1^)	log_10_(P4)	*P*_Pregnant_(%)	Lower CI (%)	Upper CI (%)	Pregnancy designation
Mn13_037A	37	2013	1.18	0.071	0.00	0.00	0.00	No
Mn16_081C-O	81	2016	1.69	0.228	0.00	0.00	0.00	No
Mn16_051D-V	51	2016	3.33	0.522	0.00	0.00	0.00	No
Mn15_019D-P	19	2015	3.93	0.595	0.00	0.00	0.00	No
Mn16_078E-O	78	2016	4.86	0.686	0.00	0.00	0.00	No
Mn14_030U	30	2014	11.81	1.07	0.15	0.00	100	Und
Mn16_098A-P	98	2016	51.17	1.709	100	100	100	Yes
Mn16_079C-O	79	2016	98.70	1.994	100	100	100	Yes
Mn15_070B	70	2015	117.75	2.071	100	100	100	Yes
Mn13_015A	15	2013	422.05	2.625	100	100	100	Yes
Mn16_089A-P	89	2016	686.03	2.836	100	100	100	Yes

## Discussion

Using a combination of field observations and biological samples, we developed a robust model to accurately assign pregnancy in free-swimming humpback whales based on progesterone concentrations from blubber biopsy samples. We observed large differences in progesterone concentration between all humpback whale females classified as pregnant and not-pregnant and smaller differences in progesterone concentrations between different demographic groups within the same pregnancy classification (e.g. among not-pregnant females and among known not-pregnant juveniles). Females that were known to be pregnant were successfully classified regardless of the timing across our wide sampling period (April through December), suggesting this method is effective at identifying even early pregnancy, once females are on their feeding ground. Finally, observed progesterone patterns were similar to those found in other cetaceans and provide further evidence supporting the use of blubber biopsy samples as an analytical matrix for assigning pregnancy in cetaceans (Clark *et al.*, 2016; Kellar *et al.*, 2013; Mansour *et al.*, 2002; Pérez *et al.*, 2011; Trego *et al.*, 2013).

Our results have also identified three potential pregnancies that could not be detected from field observations. For the three cases we report on, this was not unexpected as it would be rare to have no reproductive failures among a sample of this size (Clutton–Brock and Coulson, 2002; Kellar *et al.*, 2017); here, we specifically define “reproductive failure” as an identified pregnancy failing to produce a calf or failing to produce a calf that survives for sufficient duration to be observed given the sighting frequency and survey effort implicitly represented in the Gulf of Maine Whale Catalogue. The observed reproductive failure for this duration was 3/15 (13.3%), well within the expected rate for mammals. The specific cause of reproductive failure among both resting and lactating females was unknown, but seems to have either affected mid- to late-stage pregnancies or early life calf survival. These frequencies are consistent with the first-year humpback whale calf mortality estimates (18.2–24.1%) from the North Pacific (Gabriele *et al.*, 2001), as well as similar studies on the reproductive success of other cetaceans (Kellar *et al.*, 2017). A study on North Atlantic right whales estimated that about half of the presumed late term or young of year mortalities were of a perinatal nature (Browning *et al.*, 2010). We would expect calf mortality to be highest near the time of birth, and thus challenging to detect through observational studies. Though we believe that reproductive failure is the most likely cause for the observed high progesterone concentrations and absence of a calf the following year among these three females and that future pregnancy testing can potentially provide an upper bound on this mortality, it is important to consider the other possibilities that could lead to this outcome.

Pseudopregnancy is common among many mammals and is the process whereby the longevity of the CL and duration of elevated progesterone concentrations are prolonged, even in the absence of implantation (Bergfelt *et al.*, 2011; Robeck *et al.*, 2018). In all studied captive cetaceans, under normal conditions the CL arises during ovulation and remains active during the entire duration of the pregnancy (Robeck *et al.*, 2018). Shortly following parturition or in the event the egg is not fertilized, the CL degenerates relatively rapidly into a non-functional body the corpus albicans (Robeck *et al.*, 2018). Pseudopregnancy has been observed in several species of odontocetes (e.g. false killer whales, bottlenose dolphins, killer whales) (Atkinson *et al.*, 1999; Robeck *et al.*, 2001; 吉岡基 *et al.*, 1986) and generally occurs following several unsuccessful oestrous cycles or after early embryonic loss when maternal recognition of pregnancy has already begun (Robeck *et al.*, 2001). It has been shown in horses, that pseudopregnancy is common after mating (25%) but occurs less frequently in non-mated mares (4%) (Ginther, 1990). Work by Tarpley *et al.* (2016) noted the presence of large mature CLs among four bowhead whale ovaries even in the absence of a foetus, indicating the potential for either an early, pseudo or non-fertile pregnancy. Additionally, Robins (1954) found contrary evidence among several examined mature female humpback whales that ovulated more than once without a successful fertilization and showed signs of rapid CL regression with no signs of a prolonged CL. To our knowledge, the rate at which this anomaly might occur in wild cetaceans, and particularly baleen whales, is still unknown and thus we cannot account for this possible confounding signal. However, as it is more often linked to either embryonic loss or implantation failure it still provides information about reproductive loss. Although, if pseudopregnancy occurs at meaningful rates within wild populations it can obscure when, relative to gestation, true reproductive losses occur.

We also need to consider the timing of sampling of the three anomalous females relative to peak breeding. These females were sampled late in the feeding season (August and September), ~4–5 months prior to peak breeding. Chittleborough (1954) found that among 290 mature resting female humpback whales taken off the West Australian coast 1–2 months prior to peak breeding, 4.5% showed signs of an early CL development. Conversely, more than 90% of mature females had ovaries in the resting position during this same period. As breeding peaked and whales began to head south to feed, the proportion of females with developing CLs increased to over 80%. Though Chittleborough (1954) found evidence of an early luteal phase among a small portion of female humpbacks examined in the Southern Hemisphere, given the difference in timing relative to peak breeding between these two datasets, we consider early ovulation, in this case 5–6 months early, to be a very unlikely cause for the three observed reproductive anomalies.

We observed one female (CCS1999-092) that was simultaneously lactating and had high progesterone, but was not observed with a calf the following field season. Consecutive year calving is known to occur in humpback whales, and has been observed at a low (2%) frequency in this population (Robbins, 2007). However, the frequency at which such pregnancies occur, versus carried to term, has yet to be established for any baleen whale population. Such data would provide important new information on the mating system as well as population dynamics and recovery potential.

We developed a model that effectively and accurately predicts the probability of pregnancy in female humpback whales from populations with no demographic information. This is likely the case for the majority of other baleen whale populations around the world. However, it is important to outline how these interpretations would change given another set of assumptions, and thus, we developed three additional models and discuss their assumptions and results here. Model 1 is the model developed in this the current analysis. The three anomalous females were not included in this model, juveniles and mature pregnant and not-pregnant females were included. Model 2 encompassed all samples with the anomalous females assigned as pregnant (i.e. this assumed that the three anomalous animals where pregnant but did not produce a calf or did not produce a calf that survived for sufficient time to be observed), model 3 encompassed only sexually mature females without the anomalous females, and model 4 included all samples with the anomalous females assumed to be pseudopregnant (i.e. as if known to be non-pregnant). Models 1–3 were nearly identical and as such produced nearly identical results across the 11 WAP unknown samples, however, in the extreme case (model 4) the predicted uncertainty for the WAP unknowns increased, the probability of pregnancy at high progesterone concentrations decreased and the probability of pregnancy at low progesterone concentrations increased. For a graphical and numerical interpretation of these results, see Supplementary Fig. S1 and Table S1. We believe that model 4 is the most radical interpretation of the reference data, model 2 is the most likely interpretation of the reference data, and models 1 and 3 are scientifically conservative intermediates. The fact that numerically models 1–3 yield almost identical results provides evidence of the robustness across different interpretations of these outcomes.

A series of studies have recently used other biological sources of endocrine matrices collected by a variety of methods (faeces, blow, baleen) to determine pregnancy status in free-swimming cetaceans (Hunt *et al.*, 2014b; Kellar *et al.*, 2013; Richard *et al.*, 2017; Rolland *et al.*, 2005). Given the accuracy of our model from biopsy samples, our methods can provide additional support for interpreting the hormone signature in these other matrices, particularly when a biopsy sample can also be obtained during the secondary matrix collection. This is particularly important as Kellar *et al.* (2013) noted that the relationship between these other hormone matrices and blubber is likely not linear.

One application of the method, we have described here is to better assess both individual and population level variation in reproductive parameters, such as pregnancy, where long-term life history information currently does not exist. This situation is the norm, rather than the exception, and thus has wide-ranging value for a number of species and populations around the world. Though the relationship between hormone levels and cetacean blubber have been evaluated quite extensively in the last decade (Clark *et al.*, 2016; Kellar *et al.*, 2013, 2017, 2006; Mansour *et al.*, 2002; Pérez *et al.*, 2011), little information currently exists to link species-specific validations of these methods with other indicators of pregnancy (e.g. life history or field ultrasounds; Kellar et al., 2017).

In conclusion, this work represents the first effort to validate blubber endocrine assignments of pregnancy in free-ranging marine mammals, using humpback whales as an example. The statistical method that we employed provides a means to establish baselines or to use archived samples from many mammalian species (both marine and terrestrial) to ask questions about population change and demography. Such tools are critical, as the conservation and management of these species and populations requires accurate life history and demographic knowledge.

## Supplementary Material

Supplementary Data

Supplementary Data

## Footnotes

1 Progesterone concentrations are reported as the ng progesterone g^−1^ blubber (ng g^−1^). Endocrine pregnancy status refers to the pregnancy assignment based on the progesterone concentrations and logistic model output. Field pregnancy assignment refers to the field-observed reproduction status of the individual female when she was re-sighted the following field season. For example, a “Lactating” status refers to a female that was biopsied accompanied by a calf and observed without a calf the following field season. “Resting” refers to a female that was not accompanied by a calf when biopsied nor was it accompanied by a calf the following field season. “Pregnant” refers to a female that was biopsied and was either accompanied by a calf or not and observed with a calf the following field season. Reproductive outcome designates the combined assessment of both endocrine and field pregnancy assignments. In the reproductive outcome column, 1 = females below the minimum age at sexual maturity (juvenile), 2 = females that did not return with a calf and 3 = females that returned with a calf. Females with progesterone concentrations consistent with pregnancy that did not return with a calf are marked with an *.

## Data accessibility

The datasets supporting this article are available and archived on host institution servers, and can be accessed by contacting the corresponding author.
