# Supplementary figures and images for: Validation of a blubber-based endocrine pregnancy test for humpback whales
Source: Conserv Physiol. 2018 Jun 20;6(1):coy031. doi: 10.1093/conphys/coy031 (PMC6009693; doi:10.1093/conphys/coy031)

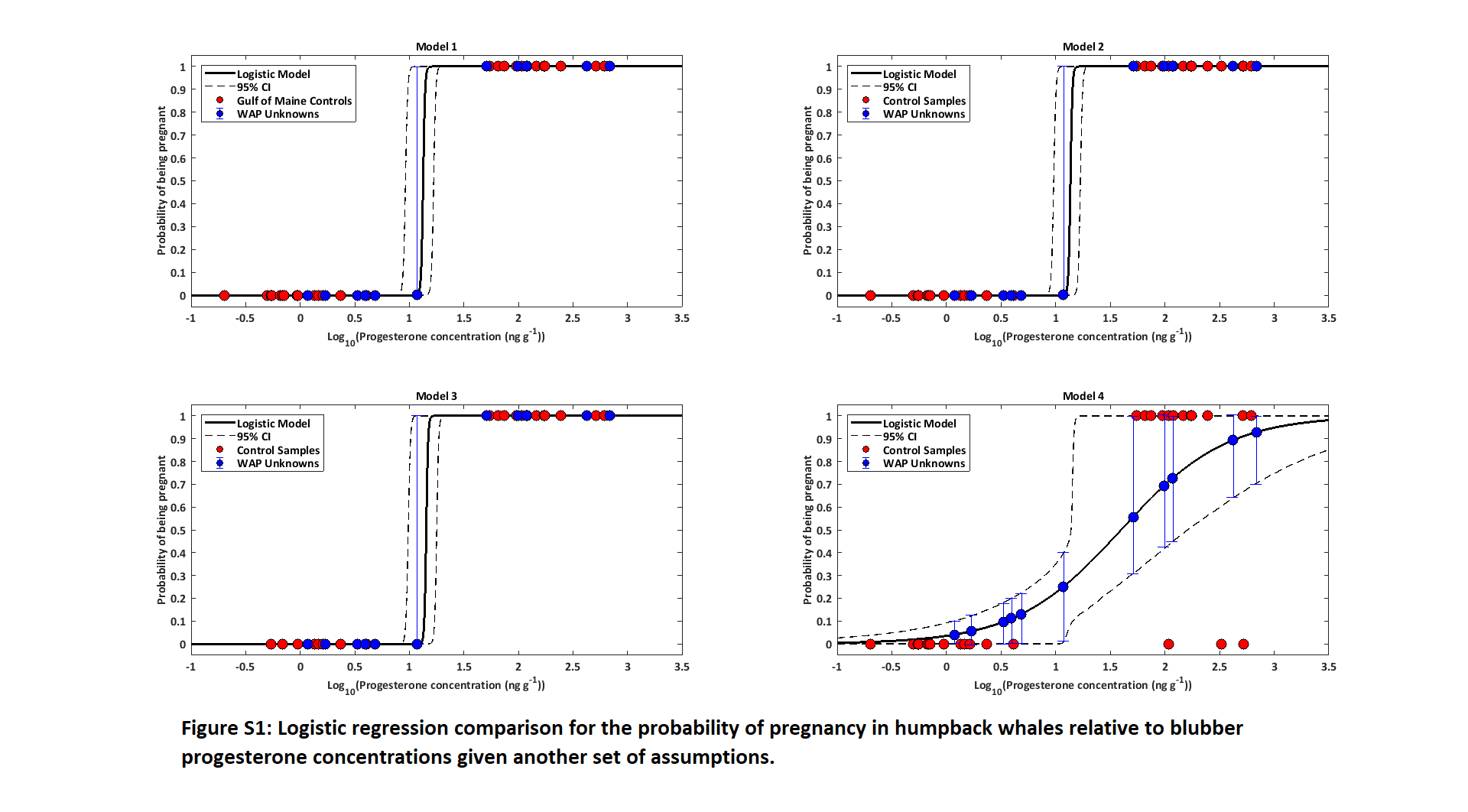

Supplement: Supplementary Data [file coy031_fig_s1.png]
