# Supplementary material for: Validation of a blubber-based endocrine pregnancy test for humpback whales
Source: Conserv Physiol. 2018 Jun 20;6(1):coy031. doi: 10.1093/conphys/coy031 (PMC6009693; doi:10.1093/conphys/coy031)
Supplement: Supplementary Data [file coy031_table_s1.docx]

Table S1: Model comparison of endocrine results and pregnancy assignments for eleven chosen female humpbacks of unknown pregnancy status sampled along the Western Antarctic Peninsula.

| **Sample ID** | **Year** | **Progesterone (ng g^-1^)** | **log_10_(P4)** | **P_Pregnant_ M 1** | **Lower CI M 1** | **Upper CI M 1** | **P_Pregnant_ M 2** | **Lower CI M 2** | **Upper CI M 2** | **P_Pregnant_ M 3** | **Lower CI M 3** | **Upper CI M 3** | **P_Pregnant_ M 4** | **Lower CI M 4** | **Upper CI M 4** |
| --- | --- | --- | --- | --- | --- | --- | --- | --- | --- | --- | --- | --- | --- | --- | --- |
| Mn13_037A | 2013 | 1.18 | 0.071 | 0.00% | 0.00% | 0.00% | 0.00% | 0.00% | 0.00% | 0.00% | 0.00% | 0.00% | 4.05% | 0.00% | 10.03% |
| Mn16_081C-O | 2016 | 1.69 | 0.228 | 0.00% | 0.00% | 0.00% | 0.00% | 0.00% | 0.00% | 0.00% | 0.00% | 0.00% | 5.52% | 0.00% | 12.28% |
| Mn16_051D-V | 2016 | 3.33 | 0.522 | 0.00% | 0.00% | 0.00% | 0.00% | 0.00% | 0.00% | 0.00% | 0.00% | 0.00% | 9.70% | 0.00% | 18.10% |
| Mn15_019D-P | 2015 | 3.93 | 0.595 | 0.00% | 0.00% | 0.00% | 0.00% | 0.00% | 0.00% | 0.00% | 0.00% | 0.00% | 11.11% | 0.00% | 19.91% |
| Mn16_078E-O | 2016 | 4.86 | 0.686 | 0.00% | 0.00% | 0.00% | 0.00% | 0.00% | 0.00% | 0.00% | 0.00% | 0.00% | 13.11% | 0.00% | 22.45% |
| Mn14_030U | 2014 | 11.81 | 1.07 | 0.15% | 0.00% | 100% | 0.67% | 0.00% | 99.80% | 0.00% | 0.00% | 99.97% | 25.06% | 0.87% | 39.74% |
| Mn16_098A-P | 2016 | 51.17 | 1.709 | 100% | 100% | 100% | 100% | 100% | 100% | 100% | 100% | 100% | 55.69% | 30.85% | 100% |
| Mn16_079C-O | 2016 | 98.70 | 1.994 | 100% | 100% | 100% | 100% | 100% | 100% | 100% | 100% | 100% | 69.41% | 41.74% | 100% |
| Mn15_070B | 2015 | 117.75 | 2.071 | 100% | 100% | 100% | 100% | 100% | 100% | 100% | 100% | 100% | 72.69% | 44.91% | 100% |
| Mn13_015A | 2013 | 422.05 | 2.625 | 100% | 100% | 100% | 100% | 100% | 100% | 100% | 100% | 100% | 89.35% | 64.08% | 100% |
| Mn16_089A-P | 2016 | 686.03 | 2.836 | 100% | 100% | 100% | 100% | 100% | 100% | 100% | 100% | 100% | 92.85% | 70.44% | 100% |
